# Dosimetric impact of intrafraction patient motion on MLC‐based 3D‐conformal spatially fractionated radiation therapy treatment of large and bulky tumors

**DOI:** 10.1002/acm2.14469

**Published:** 2024-07-19

**Authors:** Josh Misa, Alex Volk, Mark E. Bernard, William St. Clair, Damodar Pokhrel

**Affiliations:** ^1^ Department of Radiation Medicine Medical Physics Graduate Program University of Kentucky Lexington Kentucky USA

**Keywords:** bulky tumors, dosimetric impact, intrafraction motion, MLC‐based SFRT, setup error

## Abstract

**Purpose:**

To evaluate the dosimetric impact on spatially fractionated radiation therapy (SFRT) plan quality due to intrafraction patient motion via multi‐field MLC‐based method for treating large and bulky (≥8 cm) unresectable tumors.

**Methods:**

For large tumors, a cone beam CT‐guided 3D conformal MLC‐based SFRT method was utilized with 15 Gy prescription. An MLC GTV‐fitting algorithm provided 1 cm diameter apertures with a 2 cm center‐to‐center distance at the isocenter. This generated a highly heterogeneous sieve‐like dose distribution within an hour, enabling same‐day SFRT treatment. Fifteen previously treated SFRT patients were analyzed (5 head & neck [H&N], 5 chest and lungs, and 5 abdominal and pelvis masses). For each plan, intrafraction motion errors were simulated by incrementally shifting original isocenters of each field in different x‐, y‐, and z‐directions from 1 to 5 mm. The dosimetric metrics analyzed were: peak‐to‐valley‐dose‐ratio (PVDR), percentage of GTV receiving 7.5 Gy, GTV mean dose, and maximum dose to organs‐at‐risk (OARs).

**Results:**

For ±1, ±2, ±3, ±4, and ±5 mm isocenter shifts: PVDR dropped by 3.9%, 3.8%, 4.0%, 4.1%, and 5.5% on average respectively. The GTV(V7.5) remained within 0.2%, and the GTV mean dose remained within 3.3% on average, compared to the original plans. The average PVDR drop for 5 mm shifts was 4.2% for H&N cases, 10% for chest and lung, and 2.2% for abdominal and pelvis cases. OAR doses also increased. The maximum dose to the spinal cord increased by up to 17 cGy in H&N plans, mean lung dose (MLD) changed was small for chest/lung, but the bowel dose varied up to 100 cGy for abdominal and pelvis cases.

**Conclusion:**

Due to tumor size, location, and characteristics of MLC‐based SFRT, isocenter shifts of up to ±5 mm in different directions had moderate effects on PVDR for H&N and pelvic tumors and a larger effect on chest tumors. The dosimetric impact on OAR doses depended on the treatment site. Site‐specific patient masks, Vac‐Lok bags, and proper immobilization devices similar to SBRT/SRT setups should be used to minimize these effects.

## 1 INTRODUCTION

Spatially fractionated radiotherapy (SFRT) also known as GRID therapy is a single high radiation dose delivery method employed to treat unresectable, large, and bulky (≥8 cm) tumor masses while respecting skin toxicity. Historically in the orthovoltage era, SFRT was delivered with a large and heavy brass or Cerrobend physical GRID block.^1^ In the megavoltage era, clinical implementation of single‐field physical GRID block for a single fraction treatment has shown great local control for both palliative and curative intents (62.5%−91% of patient cohort), while managing toxicities to the patient, seeing no grade 3 or higher skin reaction, subcutaneous, mucosal, gastrointestinal, or central nervous system symptoms.^2^, ^3^, ^4^, ^5^, ^6^ There have been many studies investigating the mechanisms behind the high tumor response rate from the SFRT treatments. One potential mechanism is bystander signaling, in which cells dying release signaling molecules to surrounding cells which encourages neighboring cell death.^7^ Furthermore, cell death may also release effector cells such as cytokines which may induce neighborhood cell death—an effect known as intratumor immune response.^8^ The other potential mechanism may be microvasculature damage within the tumor environment due to the high spatial dose of radiation, in which endothelial cell death induces surrounding tumor cell death.^9^, ^10^, ^11^

However, the historical physical GRID block has many shortcomings. Due to the single field, it is less effective for deep‐seated tumors, and not readily available for many clinics. Because of lack of commissioning in the user's treatment planning system (TPS) it does not provide accurate dosimetric data, and the block itself is very heavy (∼25 pounds) which poses a safety concern for both the patient and radiation therapists setting up the physical block. In addition, use of a physical GRID block does not allow for pre‐treatment setup verification and correction via image‐guidance, which adds to patient positioning uncertainty for this high dose of single fraction radiation. To overcome the main concerns of the single‐field physical GRID block, we have introduced a novel method of 3D MLC‐based SFRT.^12^, ^13^ The SFRT dose distributions generated from MLC leaves have demonstrated superior dosimetry to protect adjacent critical organs to the physical block while providing better tumor response and effective management of deep‐seated tumors.^14^, ^15^ Unlike other advanced SFRT methods^16^, ^17^, ^18^, ^19^ such as inversely optimized Lattice therapy, proton SFRT, and helical tomotherapy SFRT, this 3D conformal MLC‐based method requires less physics support with no patient specific quality assurance (QA), other than routine independent second physics monitor unit (MU) calculations. Typically, these advanced methods require much longer contouring and treatment planning and are not widely accessible for remote and underserved patient cohorts. This 3D MLC crossfire technique overcomes these hurdles and facilitates same‐day SFRT treatment for both curative and palliative intents and has been demonstrated to be a safe, fast, and efficient treatment option with promising local tumor control rate and pain relief.^20^

However, like other advanced SFRT methods, a significant concern for this multi‐field MLC‐based crossfire method is the dosimetric impact of potential intra‐fraction patient motion error. Although the overall treatment time including patient setup, CBCT‐guided verification, setup corrections, and beam‐on time was within a 15‐min treatment slot, the high single dose of 15 Gy (or higher) with multiple gantry rotations (up to 6 angles), as well as the patient's tumor burden, can hinder their ability to lie still in the treatment position for the duration of the treatment. In this study, we present our simulation study to assess the dosimetric impact of intrafraction patient motion error between the treatment fields via 3D MLC‐based SFRT treatments to site‐specific bulky tumors.

## 2 METHODS AND MATERIALS

### 2.1 Patient characteristics & SFRT treatment planning

To effectively manage the complex patients with large and bulky unresectable tumors, our clinic has been utilizing a 3D conformal MLC fitting algorithm^12^, ^13^ for the past several years under an IRB approved protocol from our institution. This technique has been used to simulate, plan, and treat more than 100 extracranial patients (masses in H&N, breast, chest, abdominal, pelvis, liver, adrenal, para‐spinal, and extremity sites) on the same day as CT simulation. Site‐specific patients were set up in the supine position and immobilized either using a long H&N mask with their arms by their side, or a Vac‐Lok bag (CIVCO system, Orange City, IA) with their arms above their head. Planning CT images were acquired on a SOMATOM go.Open Pro CT scanner (Siemens Healthineers, Malvern, PA) with acquisition parameters of 512 × 512 pixels and 2.0 mm slice thickness. The 3D CT images were then imported into the Varian Eclipse TPS (Varian Medical Systems, Palo Alto, CA) where the attending physician delineated the gross tumor volume (GTV) and the adjacent OARs for dose reporting.

This technique results in high dose rods shape within the GTV. With 90° collimator rotation, this technique utilizes up to 6 coplanar MLC fields with gantry angles spaced 60° apart. Using the BEV of each field, MLCs are fitted to the GTV to have 1 cm of open leaves centered on isocenter, followed by 1 cm of closed leaves, and so forth until the entire GTV is covered by this pattern. Jaws were positioned at the edges of the apertures to minimize leakage. This method results in a distribution of high dose cylinders of 1 cm diameter with a center‐to‐center spacing of 2 cm without generating a lattice structure.^12^ By having these coplanar treatment fields equally spaced in gantry angle, the corresponding MLC positions in each gantry angle overlap to generate sieve‐like dose distribution. For dose reporting, the advanced Acuros‐based dose engine in Eclipse (Versions 15.6 and 16.2) was used with 1.25 mm dose calculation grid size. Patients were treated on C‐arm linacs equipped with an onboard CBCT imager for pre‐treatment verification. All machine‐related QA tests similar to SBRT requirements^21^ as well as independent dose verification via in‐house Monte Carlo‐based secondary MU check were completed before treating each SFRT patient.

**FIGURE 1:**
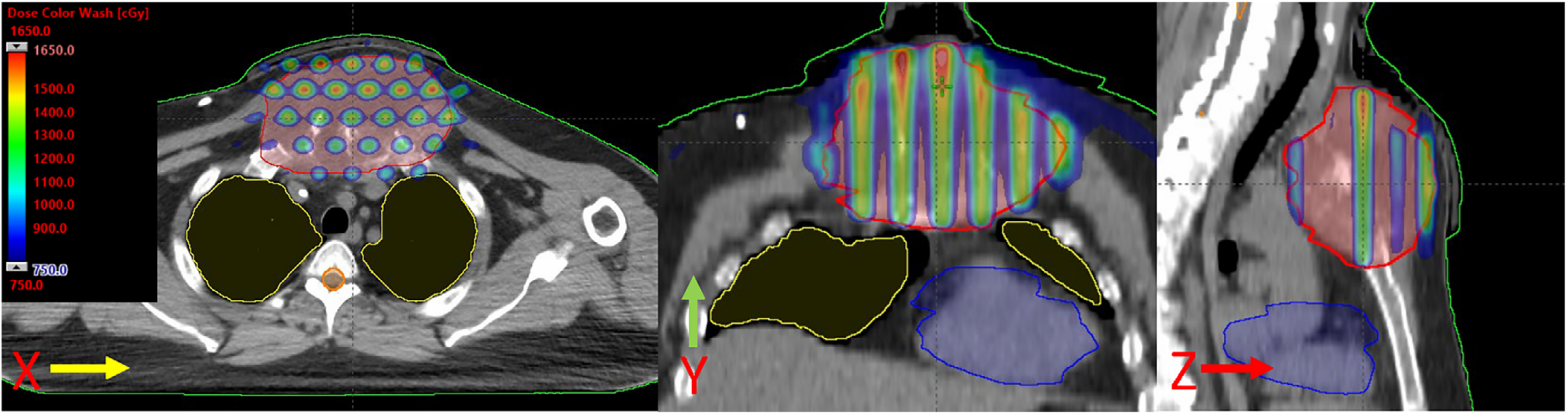
Demonstration an isodose colorwash (50% to 110%) for axial, coronal, and sagittal views (see arrows) for a very large and bulky (11.0 cm diameter) chest tumor (red contour), a colorectal adenocarcinoma primary that metastasized to a mediastinal mass, that was treated via MLC‐fitting SFRT method. Due to the medial and anterior tumor position and large patient separations, the gantry angles 150° and 210° were not used. This 4‐field geometry minimized dose to the normal lungs (yellow contour). Doses to critical organs such as spinal cord maximum (2.91 Gy), heart (0.33 Gy), and MLD (0.82 Gy) was limited.

Figure 1 represents the rod‐like cylindrical dose distribution achievable using the MLC‐based 3D crossfire method for a large chest mass. This display shows the ability of the method to produce isolated rods of higher doses within the target volume and spare nearby critical organs.

### 2.2 Simulated SFRT plans

Simulation of intrafraction motion error was performed in the Eclipse TPS. This was achieved by shifting the isocenter of each treatment field in separate directions to the other fields within the same SFRT plan. This was to imitate potential patient movement from one treatment field to the others. We analyzed a total of 15 patients previously treated with SFRT. Among this cohort, five patients were treated to H&N, five to chest and lung, and five to abdominal and pelvis areas. Currently, we also treat other sites, such as extremities and scalp, but we chose these three sites as they were the most common treatment sites from our daily clinical experience. Table 1 summarizes the patient characteristics included in this study. GTV sizes varied for H&N (131.1−979.4 cc), chest & lungs (115.2−1004.8 cc), and abdominal & pelvis (63.7−936.8 cc) cases, respectively.

**TABLE 1:** Patient characteristics of the total 15 SFRT patients included in this simulation study.

Treatment site	GTV size (cc)	No. of treatment fields	Immediately adjacent OAR
H&N	451.7 (131.1−979.4)	5.0 (4−6)	Spinal cord
Chest/lungs	439.2 (115.2−1004.8)	4.6 (4−5)	Lungs
Abdominal/pelvis	364.0 (63.7−936.8)	4.8 (3−6)	Bowel

*Note*: Each cohort analyzed consists of five patients. Average tumor size and number of treatment fields are shown. Reported parameters are mean (range).

**FIGURE 2:**
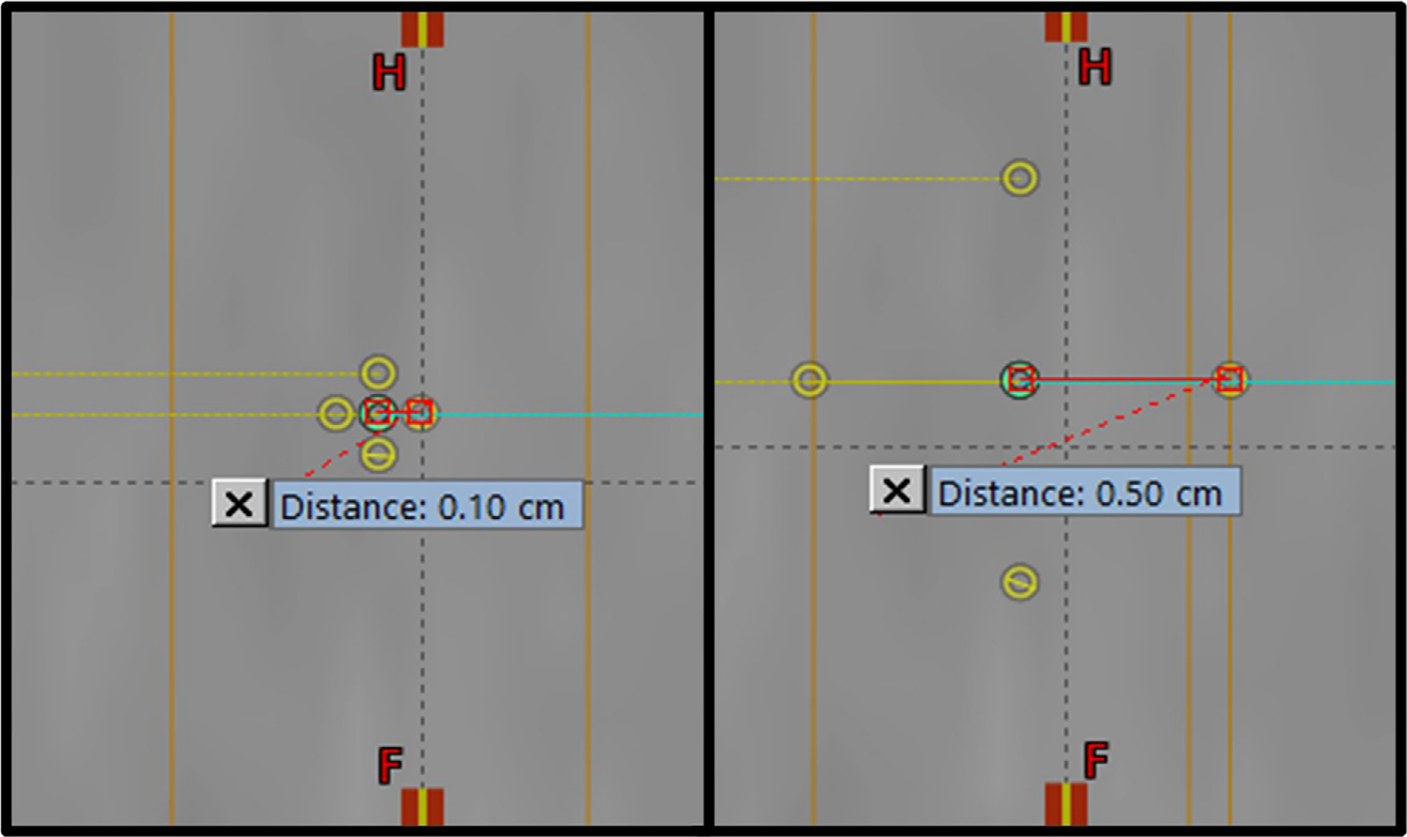
Example of a sagittal view of the isocenter shift geometry in the case of 5 mm (Right) and 1 mm (Left) shifts. Each treatment field corresponds to a different isocenter in this simulation example.

To demonstrate worst case scenarios, we chose to simulate isocenter shifts up to ±5 mm for each treatment field in each direction even though previous studies show that expected intrafraction shifts are at most 2 mm.^22^, ^23^ The reason for simulating up to 5 mm shifts was that we expected increased patient motion due to the tremendous tumor burden and pain associated with these large and bulky tumor masses undergoing SFRT treatment. Figure 2 depicts an example case of the isocenter geometry for a shifted plan. For instance, if there were 6 treatment fields, each field would be shifted in a different direction (the first field would be shifted in the −x direction, the second in the +x direction, etc. with −y, +y; −z, and +z, respectively). If there were five treatment fields used, the −x shift was skipped as the x‐direction (patient left‐right) experiences the least amount of intra‐fractional movement error as investigated by Engelsman et al.^23^ Likewise, for SFRT plans with only 4 fields used, the −z direction shift was skipped since the z‐direction (anterior‐posterior) had the second least amount of such error. By simulating one field to be positive direction, and the opposing field to be negative direction, this will simulate the worse‐case scenario, as from the crossfire method, shifting opposing fields will result in the greatest smearing of the peaks and valleys characteristic of SFRT dose distributions. Each plan had 5 simulations performed, with simulated errors of ±1 to ±5 mm shifts from the original isocenter, with 1 mm increments.

### 2.3 Data collection & analysis

For each SFRT patient cohort analyzed in the report, the dosimetric parameters reported included: PVDR = GTV(D10%)÷GTV(D90%), percentage of GTV receiving 7.5 Gy (50% of the prescription dose) GTV(V7.5), GTV mean dose, and relevant dose parameters to adjacent critical organs. Based on our previous clinical experiences, the departmental guidelines for PVDR, GTV(V7.5 Gy), mean GTV dose of a nominal prescription dose of 15 Gy are to be greater than 2.0, 50%, and 7.5 Gy, respectively. Maximum dose tolerances to site‐specific adjacent critical organs were evaluated in compliance with the single‐dose NRG‐RTOG‐0915 (Arm 1) protocol.^24^ Mean and standard deviation values for each of the dose metrics were compared for the original clinical plan versus simulated SFRT plans.

## 3 RESULTS

**FIGURE 3:**
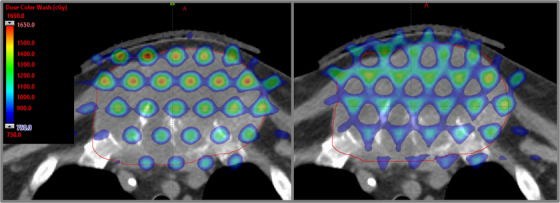
(Left) Demonstration of the axial view of the original clinical SFRT plan through the isocenter for a large chest mass (red contour) treated, as shown in Figure 1. (Right) The same slice is shown but with isocenters separated 5 mm in varying direction to extensively simulate a more extreme scenario of potential intrafractional motion error for multi‐field SFRT. The 50%‐110% isodose lines, relative to 15 Gy nominal prescription is shown.

Figure 3 depicts axial views of a chest SFRT plan with the original plan (left) and the plan with field isocenters shifted by 5 mm in varying directions (right). Visual inspection of the isodose distributions shows major changes in the isodose distributions, especially the degradation of the high‐dose regions into the low‐dose regions.

In Table 2 we show the change in resulting target PVDR, tumor coverage (GTV mean and GTV(V7.5 Gy), and corresponding OAR doses for three treatment cohorts as a function of applied isocenter shifts. Negative values indicate a decrease from the original clinical plan. For H&N dosimetric analysis for simulated shifts, less than 5% change in PVDR was observed overall. The GTV(V7.5) increased by 2.8% while mean dose increased by 8.52 cGy from the original plan. In this cohort, the maximum dose to spinal cord increased by up to 17.26 cGy in the worst‐case scenario with the largest shifts of ±5 mm. Similarly for ±5 mm shifts, chest and lungs SFRT plans had the largest decrease in PVDR of around 10% and saw corresponding decrease in GTV mean by 15.4 cGy but saw an increase in GTV(V7.5) by nearly 2.0%. The MLD changes less than 1 cGy overall with shifts. Table 2 (bottom) shows similar results for abdominal and pelvis tumors. With the largest shifts of ± 5 mm, the average PVDR fell significantly by 2.2% from the original SFRT plan, GTV(V7.5) decreased up to 4.9% and mean GTV dose decreased by 54.4 cGy. As seen in Table 2, maximum dose to bowel decreased by up to 18.2 cGy, but also increased up to 26.5 cGy depending on the magnitude of the applied shifts. Additionally, some plans had maximum bowel dose increases up to 100 cGy.

**TABLE 2:** Site‐specific dosimetric impact on SFRT plan quality due to intrafraction motion errors as simulated via isocenter shifts from the original isocenter.

H&N SFRT patient cohort [reported mean ± SD]. With original plans, PVDR = 2.7 ± 0.26; GTV mean = 7.73 ± 0.56 Gy; GTV(V7.5) = 46.3 ± 6.5 %; and maximum dose to spinal cord = 322 ± 30.1 cGy. Δ = change in metrics. Negative sign indicates less than original values.				
Applied shifts (mm)	ΔPVDR	ΔGTV mean (cGy)	ΔGTV(V7.5) (%)	ΔCord Max (cGy)
±1	−0.09 ± 0.11	−3.42 ± 12.40	0.78 ± 0.86	−4.64 ± 1.49
±2	−0.09 ± 0.13	−4.08 ± 12.37	1.40 ± 1.96	−3.62 ± 8.91
±3	−0.09 ± 0.16	−4.98 ± 11.52	2.27 ± 3.24	6.60 ± 17.39
±4	−0.10 ± 0.18	−6.54 ± 11.45	2.67 ± 3.97	11.46 ± 31.92
±5	−0.11 ± 0.21	−8.52 ± 11.03	2.81 ± 4.34	17.26 ± 39.26

Chest and lungs SFRT patient cohort [reported mean ± SD]. With original plans, PVDR = 2.5 ± 0.27; GTV mean = 7.91 ± 0.61 Gy; GTV(V7.5) = 53.1 ± 7.2 %; MLD = 69.5 ± 37.3 cGy.				
Applied shifts (mm)	ΔPVDR	ΔGTV mean (cGy)	ΔGTV(V7.5) (%)	ΔMLD (cGy)
±1	−0.10 ± 0.07	−4.76 ± 4.65	0.06 ± 0.27	0.16 ± 1.34
±2	−0.13 ± 0.08	−6.06 ± 4.84	0.44 ± 0.99	0.30 ± 1.94
±3	−0.17 ± 0.13	−9.06 ± 6.09	0.76 ± 2.25	0.42 ± 2.61
±4	−0.17 ± 0.13	−11.40 ± 7.83	1.44 ± 3.95	0.58 ± 3.32
±5	−0.25 ± 0.20	−15.40 ± 10.55	1.98 ± 5.57	0.76 ± 4.02

Abdomen & pelvis SFRT patient cohort [reported mean ± SD]. With original plans, PVDR = 2.33 ± 0.31; GTV mean = 8.14 ± 0.45 Gy; GTV (V7.5) = 53.02 ± 4.5 %; Maximum bowel = 599.6 ± 384 cGy.				
Applied shifts (mm)	ΔPVDR	ΔGTV mean (cGy)	ΔGTV(V7.5) (%)	ΔBowel Max (cGy)
±1	−0.11 ± 0.10	−6.16 ± 20.54	−0.84 ± 2.03	−14.36 ± 37.90
±2	−0.07 ± 0.14	−17.92 ± 38.99	−1.78 ± 5.25	16.66 ± 86.64
±3	−0.04 ± 0.23	−30.58 ± 60.29	−2.72 ± 8.86	26.52 ± 98.29
±4	−0.04 ± 0.28	−41.70 ± 77.64	−3.80 ± 12.00	7.60 ± 68.49
±5	−0.05 ± 0.32	−54.40 ± 96.89	−4.88 ± 14.99	−18.16 ± 45.35

*Note*: Average absolute differences are reported with SD (standard deviation) values.

It has been observed that intrafraction motion error & misalignment have moderate to severe effects on PVDR on a case‐by‐case basis, although PVDR is still being maintained above 2.0 in the worst‐case scenarios. Overall, the change in PVDR follows the expected trend of decreasing with increasing shifts, however this trend doesn't show statistical significance. Also expected, mean GTV dose decreases with increasing applied shifts by up to nearly 100 cGy. For H&N and chest & lungs plans, GTV(V7.5) exhibited an increase with increasing shifts. However, for abdominal and pelvis plans, the opposite trend was observed, where GTV(V7.5) decreases with increasing isocenter shifts applied. Overall, these GTV(V7.5) and GTV mean dose experience clinically significant changes – especially in patients going through follow‐up radiation therapy after the SFRT. Lastly, maximum dose parameters to adjacent OARs saw major effects, up to 100 cGy higher as shown for the bowel (Table 2). These changes in OAR doses may be more relevant if the patient has had previous radiation treatments, and especially for the planning of follow‐up radiation therapy. Immobilization should be used in cases where beams are entering or exiting near critical organs, to mitigate the effects of intrafraction movement errors on doses to OARs as shown here.

## 4 DISCUSSION

This study investigated the impact of intrafraction motion from this type of SFRT technique by simulating shifts between treatment fields, with the worst‐case scenario being a ±5 mm shift between treatment fields. As expected, PVDR does decrease marginally with increasing magnitude of the shift, but overall this metric stayed clinically acceptable at above 2.0. Shown in Table 2, is that the standard deviations for the PVDR increases with increasing shifts, and this trend is seen with all treatment sites. Further investigation of the data showed that the D10 of each case decreased for all cases with predictable trends, however D90 had no discernable trends which can also be seen with GTV(V7.5) values. This also demonstrated increasing standard deviation of GTV(V7.5) with an increasing applied isocenter shifts (see Table 2). Mean GTV dose decreased with increasing shifts as well. This effect is caused by the rods shape cylindrical dose distribution being shifted outside of the GTV. However, when analyzing the GTV(V7.5), the H&N and chest cases saw an increase values with increasing shifts, while the abdominal and pelvis cases gave a decrease with increasing shifts. This may be due to the pelvis cases on average having a smaller GTV volume, so the effects of intrafraction motion could be larger. This is also seen with mean GTV dose, as the pelvis cases have the largest magnitude of change compared to the other 2 common treatment sites. Maximum dose received by OAR were variable depending on the treatment site and also the magnitude of the shift applied. As expected, this may be due to the isocenter shifts causing some MLC openings in one field to move closer to the OAR, while other shifts causes MLC openings in another field to move farther away from the OAR.

Previous work has been performed on the effect of motion on the quality of SFRT plans. For instance, Ginn et al.^23^ investigated the dosimetric impact of interfraction shifts (5‐treatment scheme of lattice SFRT, as SBRT approach) due to daily patient setup uncertainty. Naqvi et al.^25^ investigated the effects of tumor motion on the PVDR by analyzing GRID distributions from a physical block onto a water phantom. A major difference in our study is that we have reported the dosimetric impact on plan quality due to the potential patient movement within a fraction via a 3D MLC‐based multi‐field SFRT method, not between fractions in multi‐fraction lattice SFRT or from a single‐field physical GRID block.

There are many clinical advantages of the 3D MLC‐based SFRT method compared to other historical and newer advanced techniques. Compared to the conventional physical GRID block, 3D MLC‐based SFRT is more appropriate for deep‐seated bulky tumors, easier to manage skin toxicity, and protects other nearby OARs. It incorporates dosimetric standards from the user's TPS and is safer for radiation therapists and patients due to the exclusion of a heavy physical block. Compared to the newer inverse‐planning‐based SFRT techniques, this 3D MLC‐based SFRT technique allows for either same‐day or next‐day treatment due to being forward‐planned with less physics validation including no need for patient‐specific IMRT QA. By allowing same‐day SFRT treatments, this will aid in pain relief for palliative intents and improve patient comfort and compliance. For therapeutic intent, this same‐day SFRT approach also helps in starting combination therapy in a timely manner while coordinating with systematic therapy for head and neck center patients. However, a limitation of this work is the number of patients included in this study and its nature as a retrospective simulation study. Many of our results have high standard deviations that may be reduced with more SFRT patients included in this study's cohort. Additionally, only translational shifts were simulated. Potential rotational intrafractional shifts may be expected as well. However, with an isocenter placed at the center of the large tumor mass, rotational shifts may not introduce large deviations compared to translational shifts. Often, patients treated with SFRT will be followed up with additional daily radiation treatments and/or have had previous radiation treatments. Sparing normal tissue organs is of high priority, especially for SFRT treatments that deliver very high doses in a single fraction to half of the tumor, spatially.

Following the recent NRG recommendations, many institutions are prospectively recruiting cancer patients with large and bulky unresectable tumors for SFRT outcome studies.^26^ To complement those recommendations for standardizing treatments for clinical trials, as shown in Table 3, we highly suggest patients receiving this method of SFRT to be simulated using appropriate immobilization devices similar to SBRT or SRT treatments to minimize the patient movement and manage PVDR, target coverage, and dose to adjacent OARs. This would include setting up site‐specific patients in an accurate and reproducible H&N SRT mask or large head and shoulder mask, an SBRT Vac‐Lok bag for abdominal and pelvis cases, adding 4DCT information for investigating tumor movement, and/or verifying patient positioning via pre‐treatment online CBCT imaging. Moreover, intrafraction motion monitoring can assist in mitigating these patient motion errors during SFRT delivery, such as utilizing surface‐guidance RT. Another way patient movement may be minimized is to reduce overall treatment times. To achieve this, we suggest treating SFRT patients using higher dose rates of at least 600 MU/min, compared to traditional physical GRID block rates of 300−400 MU/min, for these treatments our average beam on time 3.5 min. The recommendations laid out are not just limited to our MLC‐based SFRT treatments, but should also encompass advanced SFRT methods.^16^, ^17^, ^18^, ^19^ These advanced SFRT methods often have much higher MU and relatively longer treatment times, further necessitating the need for proper management of intrafraction patient motion. By ensuring accurate patient setup, minimizing patient motion, and rapid treatment delivery, target PVDR, coverage, and dose to critical organs can be managed.

**TABLE 3:** Our recommendations for same‐day MLC‐based SFRT to facilitate more accurate and reproducible patient setup & pre‐treatment verification for SFRT treatment delivery.

Patient setup devices	Long head & shoulder mask (H&N, scalp) and bolus, as needed. SBRT Vac‐Lok bag, 4D‐CT, arms above the head (thoracic & chest, breast & upper abdominal). SBRT Vac‐Lok bag, arms above the head (lower abdominal, paraspinal mass, pelvis, and thigh). Custom‐made immobilization devices (extremities).
Energy and dose rate^a^	6MV & 600 MU/min (H&N, scalp). 10MV or mixed beam & 600 MU/min (Thoracic, chest, breast, and pelvis).
Pre‐treatment patient setup verification	CBCT‐guided (use 6DOF couch correction, if available). Use kV or MV AP‐LAT Set up (if CBCT imaging cannot clear).
Intrafraction motion monitoring	Use surface‐guidance RT, if available. Use MV EPID imager “during exposure images”, capture exit fluence during SFRT delivery for offline review & documentation.

^a^ 600 MU/min is used for faster treatment delivery to minimize intrafraction patient motion error; in contrast, historically, the single‐field physical GRID block was commissioned for 300−400 MU/min.

## 5 CONCLUSION

This report demonstrates the MLC‐based SFRT treatment delivery will result in moderate to higher dosimetric discrepancies due to potential intrafraction patient movement. For a single high dose of 15 Gy SFRT, tumor coverage was degraded for patient movement errors up to ± 5 mm in any direction, and adjacent critical organs experienced changes such as small bowel maximum differing by up to 100 cGy. Thus, for more accurate treatment delivery of this method of SFRT, site‐specific patient CT simulation with appropriate immobilization devices, similar to SBRT/SRT patient setups including pre‐treatment CBCT‐guided SFRT verification, are highly recommended.

## AUTHOR CONTRIBUTIONS

Damodar Pokhrel designed the 3D MLC‐based SFRT treatment method, developed the MLC fitting algorithm, and generated clinical treatment plans. Josh Misa and Alex Volk generated simulated SFRT plans for assessing the dosimetric impact, collected, and analyzed the data. Mark E. Bernard and William St. Clair provided radiation oncology clinical expertise and supervision of the paper. Damodar Pokhrel, PhD and Josh Misa, MS drafted the preliminary manuscript. All co‐authors revised, edited, and approved the final manuscript for submission.

## CONFLICT OF INTEREST STATEMENT

The authors declare no conflicts of interest.

## Data Availability

No data is available on request due to privacy/ethical restrictions.
